# Draft genome sequences of New Delhi metallo-beta-lactamase-producing *Klebsiella pneumoniae* strains isolated from water and wastewater sources

**DOI:** 10.1128/mra.00574-25

**Published:** 2025-10-13

**Authors:** Zahraa F. Samadi, Ziad C. Jabbour, Zeinab R. Hodroj, Hadi M. Hussein, Abdallah Kurdi, Lama Hamadeh, Rami Mahfouz, Mahmoud I. Khalil, Rana El Hajj, Ghassan M. Matar, Antoine G. Abou Fayad

**Affiliations:** 1 Department of Experimental Pathology, Immunology and Microbiology, Faculty of Medicine, American University of Beirut 11238, Beirut, Lebanon; 2 Center for Infectious Diseases Research, American University of Beirut 11238, Beirut, Lebanon; 3 World Health Organization (WHO) Collaborating Center for Reference and Research on Bacterial Pathogens 66983 , Beirut, Lebanon; 4 Department of Biological Sciences, Faculty of Science, Beirut Arab University 67025, Beirut, Lebanon; 5 Department of Biochemistry and Molecular Genetics, Faculty of Medicine, American University of Beirut 11238, Beirut, Lebanon; 6 Department of Pathology and Laboratory Medicine, American University of Beirut Medical Center 66984, Beirut, Lebanon; 7 Pillar Genomics Laboratory, American University of Beirut 11238, Beirut, Lebanon; 8 Molecular Biology Unit, Department of Zoology, Faculty of Science, Alexandria University 54562, Alexandria, Egypt; Wellesley College, Wellesley, Massachusetts, USA

**Keywords:** *Klebsiella pneumoniae*, Illumina sequencing, carbapenem resistance, *bla*_NDM_ gene, Lebanon

## Abstract

Carbapenem-resistant *Klebsiella pneumoniae* is recognized by the World Health Organization as a major threat to global health. Here, we report the draft genome sequences of four New Delhi metallo-β-lactamase (NDM)-producing *K. pneumoniae* strains as part of an environmental surveillance of gram-negative bacteria isolated from various Lebanese environmental sources in 2023.

## ANNOUNCEMENT

*Klebsiella pneumoniae* is a gram-negative opportunistic pathogen able to cause hospital- and community-acquired infections (1). Carbapenem-resistant *K. pneumoniae* (CRKP) strains, particularly those producing New Delhi metallo-β-lactamase (NDM), pose a significant public health threat due to limited treatment options (2). In our study, we were able to isolate four *K*. *pneumoniae* isolates, of which KLB_111 and KLB_114 were recovered from seawater in the Antelias area of Mount Lebanon and KLB_112 and KLB_113 were obtained from raw untreated wastewater in Nabatiyeh and Beirut, respectively. Samples were enriched overnight in 6 mL autoclaved peptone water at 35°C ± 2°C, then cultured on MacConkey agar (Neogen, Michigan, USA) supplemented with 2 mg/L meropenem and incubated at 37°C. Colonies were sub-cultured and incubated overnight at 37°C in Luria-Bertani broth (Bio-Rad, California, USA) to obtain pure cultures. Taxonomic identification was initially based on colony morphology and confirmed using API 20E strips (BioMérieux, Marcy-l’Étoile, France). Antimicrobial susceptibility testing was performed using the Kirby-Bauer disk diffusion and the BioMérieux Vitek 2 compact system method in accordance with Clinical and Laboratory Standards Institute (CLSI M100Ed33) guidelines (3).

Bacterial genomic DNA was extracted from isolates cultured on MacConkey agar using the Quick-DNA Fungal/Bacterial Miniprep kit, followed by purification with the Genomic DNA Clean and Concentrator kit (Zymo Research, Irvine, CA, USA), according to the manufacturer’s protocol. For Illumina sequencing, DNA libraries were prepared using the Illumina DNA Prep kit (Illumina, San Diego, CA, USA) in combination with IDT for Illumina DNA UD Indexes (Illumina, San Diego, CA, USA), following the manufacturer’s protocol. Libraries were quantified using the Qubit dsDNA High Sensitivity Assay Kit on a Qubit 4 fluorometer (Thermo Fisher Scientific, Waltham, MA), pooled, denatured, diluted, spiked with PhiX Control v3 (Illumina, San Diego, CA, USA), and sequenced on the Illumina MiSeq platform using a MiSeq v2 Reagent Kit (500 cycles) to generate 250 bp paired-end reads, with an average coverage of ~100×. Read quality was assessed using FastQC (v0.74) (4), and trimming was performed using Trimmomatic (v0.39) (5). Cleaned reads were assembled *de novo* using Unicycler (v0.5.1) (6), and genome quality was evaluated using CheckM (v1.2.4) (7) with the “lineage_wf” workflow. Genome annotation was performed using Prokka (v1.14.6) (8). Default parameters were used for all software. Analyses were performed via the UseGalaxy platform (https://usegalaxy.org/).

Antimicrobial resistance genes were identified using CARD (Comprehensive Antibiotic Resistance Database) via the Resistance Gene Identifier tool (v6.0.1, CARD v3.2.9) (9) and AMRFinder (v3.10.5) (10). Multilocus sequence typing (MLST) was used for sequence typing (v2.19.0) (11), and PlasmidFinder (v2.1) (12) was used for plasmid detection, and taxonomic identification was confirmed through BLASTN (v2.13.0) against the NCBI nucleotide database (13).

The genome sizes of the assemblies had a median of 5.5 Mb (interquartile range [IQR] 5.3–5.9 Mb). The N50 values had a mean of 201.4 kb (SD ±93.4 kb), ranging from 135.3 kb to 284.9 kb. According to our results, the isolates were positive for several antimicrobial resistance genes, including *bla_NDM-1_*, *bla_NDM-5_*, *bla_TEM-1_*, and *bla_CTX-M-15_*. Plasmid replicons were also predicted with the presence of 13 replicons. In conclusion, the presence of CRKP in the environment highlights its role as a critical reservoir of antimicrobial resistance determinants.

**TABLE 1:** Assembly statistics of CRKP isolates and some genomic characteristics, including resistance genes and plasmid replicons

Feature	KLB_111	KLB_112	KLB_113	KLB_114
Sample accession	SAMN44756282	SAMN44756281	SAMN44756283	SAMN44756284
SRA run (SRR)	SRR31360636	SRR31360637	SRR31360658	SRR31360657
Assembly name	KLB_111	KLB_112	KLB_113	KLB_114
Assembly accession	JBQVZN000000000	JBQVZM000000000	JBQVZL000000000	JBQVZK000000000
No. reads	1,716,242	1,974,556	1,851,560	1,686,970
Genome size (bp)	5,324,647	5,926,371	5,502,508	5,540,360
Coverage (×)	65.43	77.38	34.97	80.46
No. contigs	115	106	97	74
N50	139,798	284,926	135,302	245,449
GC content (%)	57.22%	56.56%	57.27%	57.18%
Completeness (%)	97.32%	100%	100%	99.7%
Contamination (%)	0.39%	1.84%	0.50%	0.73%
Total predicted genes	5,132	5,639	5,160	5,233
Carbapenem resistance genes	*bla*_NDM-5_ and *bla*_OXA-181_	*bla*_NDM-5_	*bla*_NDM-1_	*bla*_NDM-1_
β-lactam resistance genes	*bla*_SHV-11_, *bla*_SHV-173_, *bla*_CTX-M-15_, and *bla*_TEM-1_	*bla*_SHV-11_, *bla*_CTX-M-15_, and *bla*_TEM-1_	*bla*_OXA-1_, *bla*_SHV-28_, *bla*_CTX-M-15_, and *bla*_TEM-1_	*bla*_OXA-1_, *bla*_OXA-9_, *bla*_SHV-11_, *bla*_CTX-M-15_, and *bla*_TEM-1_
Fluoroquinolone resistance	*gyrA*	*gyrA* and *parC*	*gyrA* and *parC*	*gyrA* and *parC*
Sulfonamide resistance	*sul1*	*sul1*	*sul1* and *sul2*	*sul1*
Macrolide resistance	*mphA* and *Mrx*	*mphA* and *Mrx*	*mphA* and *Mrx*	*–^a^*
Plasmid Inc groups	IncFIA(HI1), IncFII, IncR, and IncX3	IncFIB(pNDM-Mar),IncHI1B(pNDM-MAR),IncFIB(pQil), and IncFIB(pKPHS1)	IncFIB(K), IncFIB(pB171), IncFII(k), IncFII(Yp), and IncX4	IncFIB(pQil) and IncR
Col plasmids	Col(pHAD28), Col440II, and ColKP3	Col(pHAD28)	–	Col(pHAD28)

^*a*^ – indicates not applicable.

## Data Availability

Genome assembly accession numbers are listed in Table 1. The *K. pneumoniae* strain genome sequences were deposited in GenBank under BioProject PRJNA613441 with the accession numbers, SRA runs (SRR), and assembly accessions, respectively: SAMN44756281, SRR31360637, and JBQVZM000000000; SAMN44756282, SRR31360636, and JBQVZN000000000; SAMN44756283, SRR31360658, and JBQVZL000000000; SAMN44756284, SRR31360657, and JBQVZK000000000.
